# Doctors' Wills

**Published:** 1914-04-18

**Authors:** 


### Doctors' Wills.

Mr. William Henry Tomlinson, M.B., F.R.C.S., L.S.A., of Chester, lecturer and medical superintendent to the Hazel Grove Branch of the St. John Ambulance Association, late house surgeon, Oldham Infirmary, and house physician, Manchester Royal Infirmary, has left £2,963.

